# LNC-ZNF33B-2:1 gene rs579501 polymorphism is associated with organ dysfunction and death risk in pediatric sepsis

**DOI:** 10.3389/fgene.2022.947317

**Published:** 2022-09-08

**Authors:** Zhaoliang Lu, Hongyan Yu, Yufen Xu, Kaining Chen, Yueling Lin, Kun Lin, Yishuai Wang, Kaixiong Xu, Lanyan Fu, Weizhan Li, Huazhong Zhou, Bing Wei, Lei Pi, Di Che, Xiaoqiong Gu

**Affiliations:** ^1^ The School of Biomedical and Pharmaceutical Sciences, Guangdong University of Technology, Guangzhou, Guangdong, China; ^2^ Guangdong Provincial Key Laboratory of Research in Structural Birth Defect Disease, Department of Clinical Biological Resource Bank, Guangzhou Institute of Pediatrics, Guangzhou Women and Children’s Medical Center, Guangzhou Medical University, Guangzhou, China; ^3^ Medical Oncology, Panyu Central Hospital, Guangzhou, Guangdong, China; ^4^ Department of Blood Transfusion and Clinical Lab, Guangzhou Institute of Pediatrics, Guangzhou Women and Children’s Medical Center, Guangzhou Medical University, Guangzhou, China

**Keywords:** sepsis, long non-coding RNA (lncRNA), single nucleotide polymorphism, susceptibility, rs579501

## Abstract

**Background:** Sepsis is a severe systemic reaction disease induced by bacteria and virus invading the bloodstream and subsequently causing multiple systemic organ dysfunctions. For example, the kidney may stop producing urine, or the lungs may stop taking in oxygen. Recent studies have shown that long non-coding RNAs (lncRNAs) are related to the dysfunction of organs in sepsis. This study aims to screen and validate the sepsis-associated lncRNAs and their functional single nucleotide polymorphisms (SNPs).

**Result:** Unconditional multiple logistic regression based on the recessive model (adjusted odds ratio = 2.026, 95% CI = 1.156–3.551, *p* = 0.0136) showed that patients with the CC genotype of rs579501 had increased risk of sepsis. Stratification analysis by age and gender indicated that patients with the rs579501 CC genotype had higher risk of sepsis among children aged <12 months (adjusted odds ratio = 2.638, 95% CI = 1.167–5.960, *p* = 0.0197) and in male patients (adjusted odds ratio = 2.232, 95% CI = 1.127–4.421, *p* = 0.0213). We also found a significant relationship between rs579501 and severe sepsis risk (CC versus AA/AC: adjusted odds ratio = 2.466, 95% CI = 1.346–4.517, *p* = 0.0035). Stratification analysis for prognosis and number of organ dysfunctions demonstrated that the rs579501 CC genotype increased non-survivors’ risk (adjusted odds ratio = 2.827, 95% CI = 1.159–6.898, *p* = 0.0224) and one to two organs with dysfunction risk (adjusted odds ratio = 2.253, 95% CI = 1.011–5.926, *p* = 0.0472).

**Conclusion:** Our findings showed that the lnc-ZNF33B-2:1 rs579501 CC genotype increases the susceptibility to sepsis. From the medical perspective, the lnc-ZNF33B-2:1 rs579501 CC genotype could be serving as a biochemical marker for sepsis.

## Background

When children are infected by pathogens, the immune system will start to attack the origin of the infection. The immune system releases chemokines into the bloodstream to fight the bacterial or viral infection; these chemokines can also attack normal organs and tissues, and this immune overreaction is called sepsis, which causes inflammation, blood flow problems, low blood pressure, trouble breathing, vital organ failure, and can even be life-threatening (Mathias et al., 2016). Sepsis in newborns and children is always caused by bacteria in the blood. Common culprits include group B *Streptococcus*, *Escherichia coli*, *Listeria monocytogenes*, *Neisseria meningitis*, *Streptococcus pneumoniae*, *Haemophilus influenzae* type B, and *Salmonella*. Sepsis is the major cause of admissions to neonatal intensive care units (NICUs) and pediatric intensive care units (PICUs) and of death (McDonald et al., 2012; Dickson et al., 2016). Recently, research has reported that 17% of worldwide mortality is associated with sepsis and that 26% of hospital mortality is due to severe sepsis (Fleischmann et al., 2016). In the United States alone, the incidence of sepsis is about 0.3% of the population; almost 72,000 children were hospitalized for sepsis, with a 25% mortality rate, throughout 2013–2014 (Balamuth et al., 2014; Ruth et al., 2014; Weiss et al., 2015). Wang et al. (2014a) reported that in Huai’an, Jiangsu, China, the incidence of sepsis among children was nearly 0.18%, and the overall case fatality rate for sepsis was 3.5%. They estimated a minimum annual incidence of more than 360,000 cases of pediatric sepsis in China. Therefore, early diagnosis and prognosis of sepsis are essential for clinical therapy.

There is overwhelming evidence supporting that sepsis is a highly heterogeneous disease with large inter-individual differences in the disease course and genetic factors influencing individual vulnerability to, and the severity of, infections (Frodsham and Hill, 2004). For example, the genetic variant rs2737190 is in the promoter region of the *TLR4* gene, and the GG genotype produces an improvement in the immune response (Colin-Castro et al., 2021). Another example is that 593C>T GPx1 SNP in sepsis patients leads to high organ dysfunction, sepsis shock, and mortality risk (Majolo et al., 2015). LncRNAs are typically defined as transcripts longer than 200 nucleotides with little or no protein coding potential (Fatica and Bozzoni, 2014). Long non-coding RNALnc (RNAs) may regulate gene expression at epigenetic, transcriptional, and posttranscriptional levels. Furthermore, single nucleotide polymorphisms (SNPs) may alter the function of lncRNAs and effect susceptibility to disease. In the case of *PACT1*, for instance, rs2632159 polymorphism could increase colorectal cancer risk (Yang et al., 2019). Whether SNPs in lncRNAs can be biomarkers for sepsis susceptibility remains unclear. Several studies have reported that some lncRNAs, such as those in *SOX2OT*, *CCAT2*, and *MALAT1* gene polymorphism, may be associated with an increased risk of sepsis (Chen et al., 2018; Wu et al., 2020; Wu et al., 2021). Lnc-ZNF33B-2:1, also known as *LOC283820/AL022334.7-001/NR_136644.1/ENST00000568976*, rs579501 located on chromosome chr10: 43246795 (GRCh37. p13), is a functional SNP in the *LOC203828* noncoding region. Furthermore, one publication has reported the association of rs579501 with gastric cancer (Duan et al., 2018).

This research aims to evaluate the relationship between the candidate lnc-ZNF33B-2:1 allele and sepsis susceptibility and to assess the effect of sepsis-associated rs579501 on the susceptibility to sepsis in the southern Chinese population to better understand the public health risk.

## Materials and methods

### Study population

We recruited 474 sepsis patients from the PICU and 678 healthy controls who visited the hospital for health checks at the Guangzhou Women and Children’s Medical Center in southern China from December 2015 to December 2019 and who presented without any other diseases.

Inclusion criteria for the case group were as follows: 1) children with clinical sepsis in the PICU of Guangzhou Women and Children’s Medical Center; 2) time range November 2015 to November 2019; 3) the subjects of this study belonged to the southern area of China based on the children’s place of origin; 4) the medical records of sick children were complete, including complete medical histories; and 5) the parents of the children provided signed informed consent before the study.

Inclusion criteria for the control group were as follows: 1) healthy children were recruited from the Department of Physical Examination in Guangzhou Women and Children’s Medical Center, with no previous history of sepsis; 2) time rage November 2015 to November 2019; 3) the subjects of this study belonged to the southern area of China, based on the children’s place of origin; 4) the medical records of control children were complete, including complete medical histories; and 5) the parents of the children provided signed informed consent before the study.

Exclusion criteria were as follows: 1) past or present history of malignant tumors or genetic diseases; 2) the description provided by parents was not clear; 3) the subjects of this study did not belong to the southern area of China; and 4) the family did not consent to the study.

Diagnostic criteria for sepsis, severe sepsis, and septic shock were based on the international definition (Goldstein et al., 2005). Sepsis describes a syndrome that occurs when severe infection leads to severe illness and effects. Severe sepsis occurs when a bacterial, viral, or fungal infection causes a significant response from the body’s immune system, causing a high heart rate, fever, or shortness of breath.

Septic shock is the most severe form of sepsis, in which underlying circulatory and cellular metabolism abnormalities are severe enough to significantly increase mortality (Singer et al., 2016).

However, the variation between different in-patient children is larger (high inter-individual variation). Based on the related pediatric sepsis literature (Dellinger et al., 2013; Weiss et al., 2015; Emr et al., 2018), the specific criteria are as follows.

Sepsis: 1) The correct evidence of the pathogen was achieved according to the routine laboratory culture identification or a highly suspected pathogen infection by clinical and imaging methods and had to include abnormal body temperature or abnormal white blood cell count. 2) Body temperature greater than 38.5°C or less than 36°C. 3) Immature neutrophil proportion >10%.
Severe sepsis: 1) Children with tachycardias due to insufficient recirculating perfusion. 2) Decreased peripheral pulse rate, capillary filling lasted for longer than 2 s. 3) Red streaks on the limbs. 4) Reduced urine volume. 5) Pediatric acute respiratory distress syndrome. 6) Two or more organ dysfunctions.
Septic shock: 1) Severe sepsis with hypoperfusion. 2) Meeting the criteria for hypotension in children at this age and severe vasodilation and hypotension refractory to aggressive fluid resuscitation. 3) Needing vasoactive drugs to maintain hemodynamic stability.

### DNA extraction and genotyping

We genotyped SNP (lnc-ZNF33B-2:1 rs579501 was purchased from Applied Biosystems) from genomic DNA isolated from venous whole blood samples of sepsis patients and healthy controls. The DNA extraction and genotyping procedures have been published previously (Wang et al., 2019).

The patients’ specimens for this study were stored in the ultra-low temperature freezer of our hospital’s clinical biobank. In addition, we quality controlled the DNA samples using the DNA electrophoresis technique: DNA samples were measured using a UV spectrophotometer, and the ratio of absorbance (OD260/OD280) was between 1.6 and 1.8.

### Statistical analysis

Initially, we examined the Hardy–Weinberg equilibrium (HWE) of the samples using SAS software (version 9.1; SAS Institute, Cary, NC). Next, a chi-squared (*χ* ^2^) test was employed to assess the significance of differences between patients and healthy controls in the frequency distributions and genotypes. To evaluate the associations between rs579501 and sepsis risk, multivariate logistic regression was used to compute odds ratios (ORs) and corresponding 95% confidence intervals (CI), adjusted for age, gender, sepsis subtype, prognosis, and number of organs with dysfunction. The statistical analysis procedures have been described previously (Lu et al., 2019).

### Data source

The gene expression datasets analyzed in our study were obtained from the GEO database (https://www.ncbi.nlm.nih.gov/geo/). Data of a total of 10 sepsis children and 12 health controls were retrieved from the database. GSE145227 was based on the Agilent GPL23178 platform ([OElncRNAs520855F] Affymetrix Human Custom lncRNA Array). All the data were freely available online, and this study did not involve any experiment on humans or animals performed by any of the authors.

### Ethics statement

The present study was approved by the Guangzhou Women and Children Medical Center Ethics Committee (2015042202) and was conducted according to the International Ethical Guidelines for Research Involving Human Subjects stated in the Declaration of Helsinki. The children’s families provided written informed consent.

## Results

### Comparison of rs579501 A/C polymorphism in southern Han Chinese population with different regional and ethnic groups

The rs579501 A/C genotypes and alleles of CHS (southern Han Chinese) population were compared with the SNP distribution data of CHB (Beijing Han Chinese), AFR (all African individuals), AMR (all American individuals), ASN (all East Asian individuals), ASW (Americans of African ancestry in SW United States), CLM (Colombians from Medellin, Colombia), and EUR (all European individuals) populations reported in the 1000 Genomes Project Database (http://asia.ensembl.org/info/docs/tools/index.htm). We found that the rs579501 CC genotype and allele of the CHS population, compared to AFR, AMR, ASW, CLM, and EUR populations, have a statistically significant difference (*p* value <0.05) (Table 1)**.**

**TABLE 1:** Sepsis patients have higher lnc-ZNF33B-2:1 level (from the GEO database).

Race	Count	Genotype			χ^2^	*p*	Allele		χ^2^	*p*
		AA	AC	CC			A	C		
CHS	100	55 (55.00)	42 (42.00)	3 (3.00)			152 (76.00)	48 (24.00)		
CHB	97	62 (63.92)	31 (31.96)	4 (4.12)	2.1741	0.3372	155 (79.89)	39 (20.110	0.6571	0.4173
AFR	246	179 (72.76)	62 (25.20)	5 (2.04)	**10.2782**	**0.0058**	420 (85.37)	72 (14.63)	**8.0612**	**0.00482**
AMR	181	174 (96.13)	7 (3.87)	0 (0.00)	**72.5151**	**<0.0001**	355 (98.06)	7 (1.94)	**68.5723**	**<0.0001**
ASN	286	176 (61.54)	101 (35.32)	9 (3.14)	1.4283	0.4896	453 (79.20)	119 (20.80)	0.7153	0.3982
ASW	61	45 (73.77)	16 (26.23)	0 (0.00)	**6.5949**	**0.0369**	106 (86.89)	16 (13.11)	**4.9752**	**0.0261**
CLM	60	58 (96.67)	2 (3.33)	0 (0.00)	**31.4062**	**<0.0001**	118 (98.33)	2 (1.67)	**26.7062**	**<0.0001**
EUR	379	377 (99.47)	2 (0.53)	0 (0.00)	**176.872**	**<0.0001**	756 (99.73)	2 (0.27)	**175.4651**	**<0.0001**

Comparison of lnc-ZNF33B-2:1 rs579501 A/C gene and allele frequency between the southern Han Chinese population and different ethnic groups [*n* (%)].

χ^2^ test for distributions between the southern Han Chinese population and different ethnic groups.

Abbreviations: CHS, southern Han Chinese; CHB, Beijing Han Chinese; AFR, all African individuals; AMR, all American individuals; ASN, all east Asian individuals; ASW, Americans of African ancestry in SW USA; CLM, Colombians from Medellin, Colombia; EUR, all European individuals.

The meaning of the bold values is that difference of groups has statistical significance (*p*-value <0.05).

### Population characteristics

Table 2 shows the demographic characteristics of sepsis patients and healthy controls. In total, 474 pediatric patients with sepsis and 678 healthy controls were included in our research. The average age of sepsis patients was 35.04 months (±34.26; range: 1–180) and 35.53 ± 29.37 months (range: 1–168) for controls. A total of 63.5% of sepsis patients were male, and the male ratio was 58.85% in the controls. The distribution of age (*p* = 0.1811) and gender (*p* = 0.111) was not significantly different between sepsis patients and controls. Among the sepsis patients, 98 children were diagnosed clinically as having sepsis, 291 children as having severe sepsis, and 85 as having septic shock. According to the number of dysfunctional organs, 276 children had damage to one to two organs, and 95 children had three or more dysfunctional organs. Ultimately, 80 children suffering from sepsis died.

**TABLE 2:** Frequency distribution of selected characteristics in sepsis cases and healthy controls.

Variable	Cases (*n* = 474)		Controls (*n* = 678)		*p* ^a^
	No.	%	No.	%	
Age range (months)	1–180		1–168		
Mean ± SD	35.04 ± 34.26		35.53 ± 29.37		0.1811
Median	25		33		
Interquartile range	10–52		13–45		
≤60	403	85.02	595	87.76	
>60	71	14.98	83	12.24	
Gender					
Male	301	63.5	399	58.85	0.111
Female	173	36.5	279	41.15	
Sepsis subtypes					
Sepsis	98	20.68	NA		
Severe sepsis	291	61.39	NA		
Septic shock	85	17.93	NA		
Prognosis					
Survivors	394	83.12	NA		
Non-survivors	80	16.88	NA		
Number of organs with dysfunction, *n* (%)					
1–2	276	74.39	NA		
3 or more	95	25.61	NA		
Source of infection					
Lung infection	278	58.65	NA		
Brain infection	36	7.59	NA		
Primary bloodstream infection	35	7.38	NA		
Abdominal infection	28	5.91	NA		
Respiratory infection	18	3.8	NA		
Urinary tract infection	8	1.69	NA		
Others	71	14.98	NA		

a Two-sided *χ* ^2^ test for distributions between sepsis cases and controls.

### Association between the lnc-ZNF33B-2:1 rs579501 A/C polymorphism and the risk of sepsis

Table3 shows the genotype distributions of lnc-ZNF33B-2:1 rs579501 A/C polymorphism in the sepsis patients and healthy controls. The lnc-ZNF33B-2:1 rs579501 A/C genotype distribution analysis used to assess the HWE in the healthy control group revealed an equilibrium (HWE = 0.149). The genotype analysis of the lnc-ZNF33B-2:1polymorphism revealed a remarkable difference in the carriers of the rs579501 genotypes exhibiting the CC allele (adjusted odds ratio = 2.026, 95% CI = 1.156–3.551, *p* = 0.0136), suggesting that rs579501 CC genotypes increased sepsis susceptibility.

**TABLE 3:** Genotype frequency distribution of lnc-ZNF33B-2:1 in sepsis cases and healthy controls.

Genotype	Cases (*n* = 474)	Controls (*n* = 678)	*p*-value^a^	OR (95% CI)	*p*-value	Adjusted OR (95% CI)	*p*-value^b^
lnc-ZNF33B-2:1/rs579501 A>G (HWE = 0149)							
TT	284 (59.92)	424 (62.54)	**0.0334**	1.000		1.000	
TG	159 (33.54)	232 (34.22)		1.023 (0.796–1.316)	0.8583	1.018 (0.791–1.310)	0.8876
GG	31 (6.54)	22 (3.24)		**2.104 (1.194–3.707)**	**0.0101**	**2.039 (1.155–3.600)**	**0.014**
Dominant	190 (40.08)	254 (37.46)	0.3687	1.117 (0.878–1.421)	0.3684	1.107 (0.870–1.410)	0.4072
Recessive	443 (93.46)	656 (96.76)	**0.0093**	**2.087 (1.192–3.651)**	**0.01**	**2.026 (1.156–3.551)**	**0.0136**

a χ^2^ tests were used to determine differences in genotype distributions between the children with KD and the controls.

b Adjusted for age and gender.

The meaning of the bold values is that difference of groups has statistical significance (*p*-value <0.05).

### Stratification analysis

The stratified analyses were based on a recessive model by age (<12 months, 12–60 months, and >60 months), gender (male vs. female), sepsis subtypes (sepsis, severe sepsis, and septic shock), prognosis (survivors vs. non-survivors), number of organs with dysfunction (one to two vs. three or more), and adjusted for other factors. We found that the rs579501 CC allele was more predominant for children aged <12 months (adjusted odds ratio = 2.638, 95% CI = 1.167–5.960, *p* = 0.0161). The CC allele was also found to be a risk factor in male patients (adjusted odds ratio = 2.232, 95% CI = 1.127–4.421, *p* = 0.0182), those with severe sepsis (adjusted odds ratio = 2.466, 95% CI = 1.346–4.517, *p* = 0.0021), non-survivors (adjusted odds ratio = 2.827, 95% CI = 1.159–6.898, *p* = 0.0198), and patients with one to two organs with dysfunction (adjusted odds ratio = 2.253, 95% CI = 1.201–4.227, *p* = 0.0078) (Table 4).

**TABLE 4:** Stratification analysis of susceptibility in sepsis patients.

Variable	AA/AC	CC	*p*-value	OR (95% CI)	*p*-value	Adjusted OR (95% CI)	*p*-value^a^
	Patients/controls						
Age (months)							
<12	214/275	19/9	**0.0125**	**2.713 (1.203–6.116)**	**0.0161**	**2.638 (1.167–5.960)**	**0.0197**
12–60	183/313	11/10	0.1582	1.881 (0.784–4.516)	0.1571	1.863 (0.776–4.475)	0.164
>60	46/68	1/3	0.5254	0.493 (0.050–4.885)	0.5454	0.497 (0.050–4.938)	0.551
Gender							
Male	278/385	23/14	**0.0161**	**2.275 (1.150–4.500)**	**0.0182**	**2.232 (1.127–4.421)**	**0.0213**
Female	165/271	8/8	0.3325	1.642 (0.605–4.460)	0.3303	1.657 (0.609–4.507)	0.3226
Sepsis subtypes							
Sepsis	95/656	4/22	0.6889	1.256 (0.424–3.723)	0.6814	1.264 (0.426–3.750)	0.673
Severe sepsis	267/656	23/22	**0.0024**	**2.568 (1.407–4.686)**	**0.0021**	**2.466 (1.346–4.517)**	**0.0035**
Septic shock	81/656	4/22	0.5044	1.473 (0.495–4.380)	0.4866	1.455 (0.488–4.339)	0.5012
Prognosis							
Survivors	370/656	24/22	0.0296	1.934 (1.070–3.497)	0.0291	1.838 (1.014–3.332)	0.0448
Non-survivors	73/656	7/22	**0.0327**	**2.860 (1.181–6.925)**	**0.0198**	**2.827 (1.159–6.898)**	**0.0224**
Number of organs with dysfunction, *n* (%)							
1–2	256/656	20/22	**0.0089**	**2.330 (1.250–4.342)**	**0.0078**	**2.253 (1.201–4.227)**	**0.0114**
3 or more	88/656	7/22	0.073	2.372 (0.985–5.714)	0.0541	**2.448 (1.011–5.926)**	**0.0472**

a Adjusted for age and gender.

The meaning of the bold values is that difference of groups has statistical significance (*p*-value <0.05).

### Power calculations

We used the online software for calculating the statistical power for rs579501 (https://zzz.bwh.harvard.edu/cgi-bin/cc2k.cgi) with the following parameters. The sample size was 474 patients and 678 controls. The minor allele frequency of rs579501 was 0.2331. The prevalence of the disease was set as 0.18%. The genotype relative risks (GRRs) for TG and GG versus TT were approximately estimated as their corresponding OR values (1.023 and 2.104, respectively). Given the threshold of the type I error rate of 0.05, the statistical power for rs579501 was 0.856 in the study.

### Prediction of lnc-ZNF33B-2:1 polymorphism centroid secondary structure and target microRNAs

**FIGURE 1:**
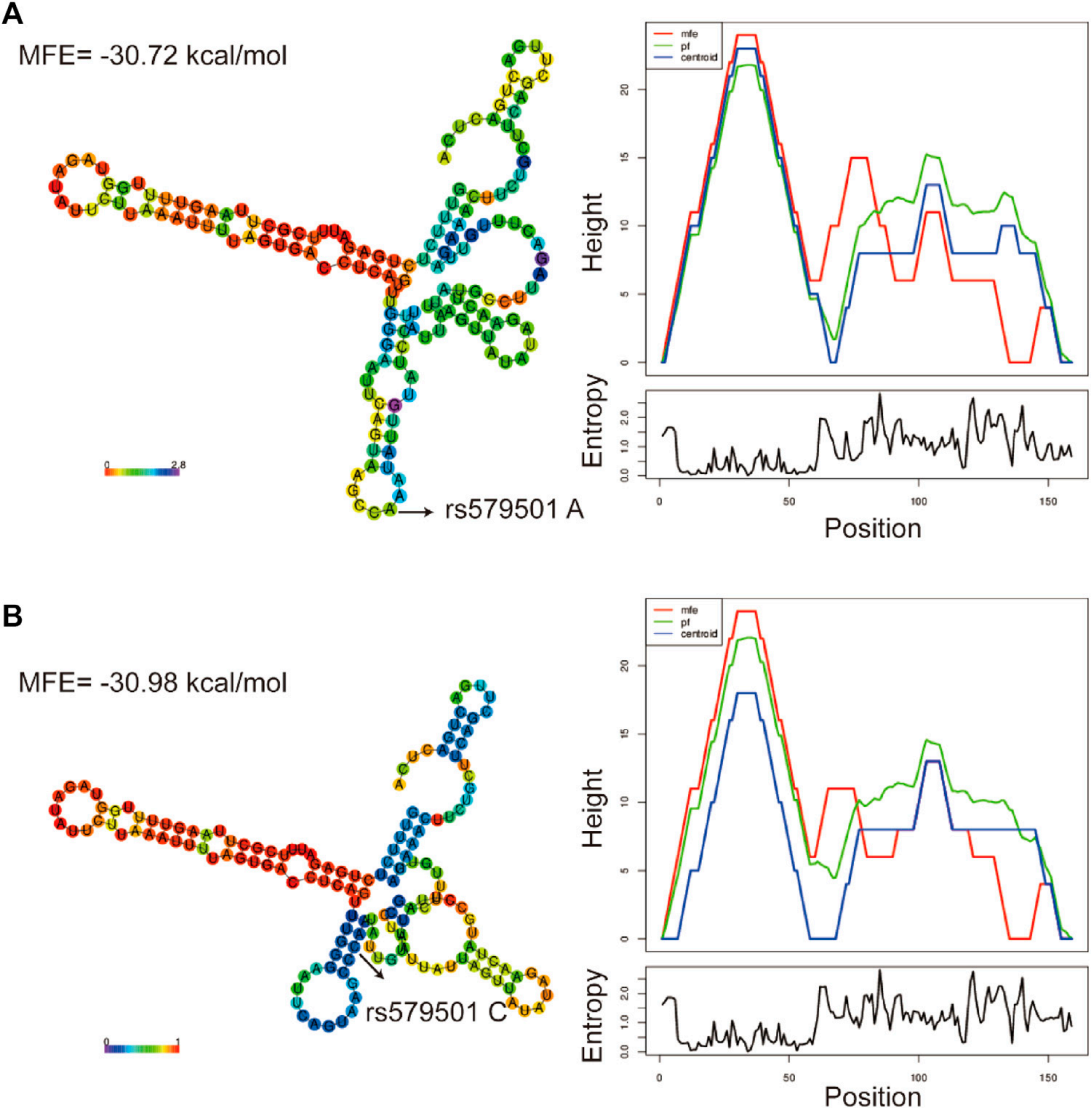
Bioinformatic prediction of lnc-ZNF33B-2:1 polymorphism on centroid secondary structure. **(A)** Centroid secondary structure and a mountain plot representation of the MFE structure of rs579501 A allele; **(B)** centroid secondary structure and a mountain plot representation of the MFE structure of rs579501 C allele.

**FIGURE 2:**
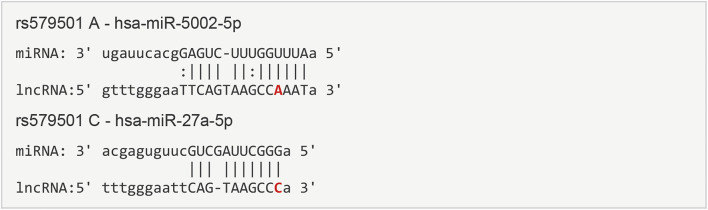
Prediction target microRNAs of lnc-ZNF33B-2:1 polymorphism. The sequence and putative binding sites of miR-5002-5p and miR-27a-5p on the different re579501 allele were validated using the lncRNA-binding prediction software program.

**FIGURE 3:**
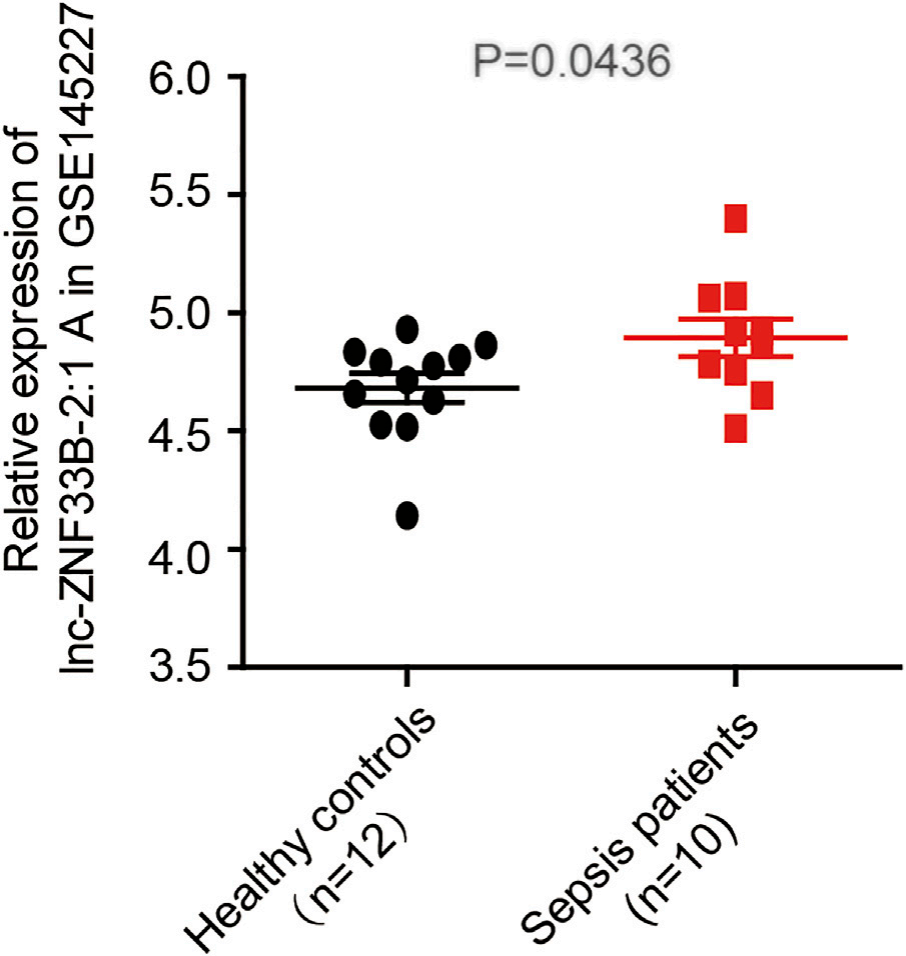
lnc-ZNF33B-2:1 expression level in sepsis and healthy control samples (data from GSE145227), revealing that lnc-ZNF33B-2:1 shows significantly higher expression in sepsis patients (*n* = 10) than normal healthy donors (*n* = 12).

The LNCipedia web server (https://lncipedia.org/) was used to find lnc-ZNF33B-2:1 rs579501 A and rs579501 C allele nucleotide sequences (Supplementary Table S1). The RNAfold web server (http://rna.tbi.univie.ac.at//cgi-bin/RNAWebSuite/RNAfold.cgi) was used for the analysis of the prediction of lnc-ZNF33B-2:1 secondary structure, including rs579501 A and rs579501 C alleles. Consequently, RNAfold prediction showed that the centroid secondary structure was markedly changed with rs579501 A>C alleles (Figure 1). The minimum free energy (MFE) of rs579501 A>C alleles was changed from –30.72 kcal/mol to –30.98 kcal/mol. By using the lncRNA-binding prediction software program (http://bioinfo.life.hust.edu.cn/lncRNASNP2/), we found that the conversion of A>C in the rs579501 polymorphism may create a binding site for has-miR-27a-5p and lead to a loss of has-miR-5002-5p binding (Figure 2). The miRBD web server (http://www.mirdb.org/mirdb/index.html) was used to predict the targets of miR-27a-5p as some proteins have been reported to be associated with aggravation of the sepsis state, such as IL-1 and gasdermin A (Supplementary Table S2). We also found that sepsis patients have a higher lnc-ZNF33B-2:1 level from the GEO database (Figure 3).

## Discussion

Genetic risk factors play an important role in the pathogenesis of sepsis. In our case–control study, we investigated the associations between lnc-ZNF33B-2:1 gene polymorphism and sepsis risk in a southern Chinese population. We found that the rs579501 CC genotype was associated with an increased risk of sepsis in children and was a risk factor in male patients and children aged 0–12 months. Furthermore, the stratified analysis revealed that the rs579501 C allele was a risk factor during severe sepsis. Moreover, patients with the rs579501 CC genotype were more susceptible to death and one to two organs with dysfunction, which were caused by sepsis. Interestingly, there were significant ethnic differences between CHS and other regions and populations in genotype or allele frequencies of rs579501 CC genotypes, with a higher proportion among the CHS population. This study shows that the lnc-ZNF33B-2:1 rs579501 CC genotype was associated with an increased risk of sepsis in the CHS population. The MFE structure of a sequence is the secondary structure that is calculated to have the lowest value of free energy. The lower the free energy, the more likely, in theory, the structure will form and the more stable it will be (Wuchty et al., 1999). In our study, we found that the lnc-ZNF33B-2:1 CC allele had the lower MFE, suggesting that the structure of lnc-ZNF33B-2:1 CC is more stable.

At first, it remained unclear how these lnc-ZNF33B-2:1 polymorphisms affect sepsis. By using the lncRNA-binding prediction software program, we found that after change rs579501 A>G, has-miR-27a-5p could functionally substitute for has-miR-5002-5p and combine with lnc-ZNF33B-2:1. The has-miR-27a-5p gene is located on chromosome 19 in humans, and a growing body of research has revealed that the miR-27a-5p gene is involved in sepsis progression. In the sepsis-induced lung injury model, this could increase the miR-27a-5p expression level, and in an LPS-reduced septic mouse model, cell infiltration was attenuated by the intratracheal instillation of the miR-27a-5p inhibitor and alleviated the inflammatory phase of sepsis (Younes et al., 2020). Furthermore, Wang et al. (2014b) reported that miR-27a was upregulated and promoted inflammatory response in sepsis. But we were still unclear whether different miRNA bindings to lnc-ZNF33B-2:1 have distinct consequences. Therefore, we tried to explore the underlying mechanism in the lncRNAs-miRNA axis function in sepsis. GSDMA was predicted to be a target protein of miR-27a-5p by the miRNA target gene prediction website. GSDMD is the final common effector of inflammasome activation, forming membrane pores to enable pro-inflammatory cytokine release and pyroptosis. GSDMD could mediate LPS-induced septic myocardial dysfunction and drive tissue injury in lethal polymicrobial sepsis (Chen et al., 2020; Dai et al., 2021). Hu identified a potent inhibitor of GSDMD pore formation and protected against sepsis (Crunkhorn, 2020). Therefore, we speculated that the rs579501 CC-miR-27a-5p-GSDMD pathway is involved in sepsis progression. Further studies will be necessary to validate these concepts. Some limitations of this study should be noted. First, in the aforementioned studies, we also found the rs579501 CC genotype in a higher proportion of CHB population. Owing to this research was subjected to geographical factors, we only analyzed the population in southern China; thus, this study lacks regional and ethnic comparisons. Second, this study also lacks dynamic monitoring of miR-27a-5p and GSDMD levels during the follow-up period.

## Conclusion

In conclusion, the present study demonstrated that the lnc-ZNF33B-2:1 rs579501 C variant is a risk factor for sepsis in southern Chinese children. The risk effect was reflected more substantially in children aged <12 months, survivors, and male patients. Moreover, the risk effect was found more in the sepsis subgroup than in the severe sepsis subgroup. In this study, we sought to identify sepsis-associated lncRNAs as potential biomarkers for diagnosis and therapy and uncovered important clues for further study of the function and mechanism of lncRNAs in sepsis. Our study illustrates that the lncRNAs polymorphism was associated with the susceptibility of sepsis. Extensive functional research and additional well-designed population-based prospective studies with different ethnic groups are warranted to confirm and extend our findings.

## Data Availability

The datasets used and/or analysed during the current study are available from the corresponding author on reasonable request.
