# Supplementary material for: LNC-ZNF33B-2:1 gene rs579501 polymorphism is associated with organ dysfunction and death risk in pediatric sepsis
Source: Front Genet. 2022 Sep 8;13:947317. doi: 10.3389/fgene.2022.947317 (PMC9678184; doi:10.3389/fgene.2022.947317)
Supplement: Supplementary file 1 [file DataSheet1.docx]

Supplement table 1: The nucleotide sequence of rs579501 A>C alleles.

| The nucleotide sequence of rs579501 A>C alleles |  |
| --- | --- |
| >rs579501 A  TCCTCAAGACTAGAAAGCATGAAGCAGGGGAGGCGTTTTGAAAGCTTAAGGACAAAAGACCATGGGCCTGGATGGCTGATTCCGGGATCGGCATCGTAATTTGTTGAGAAGGAGGCCGTGCTGTGCTGCCAGTTATTAATGGGTTTAATCGGTTGATACACAGCCCTACTGGCCTAACCAGTAGCCCAGGGCCCGAGAGGATTTGCAGGTCGTGTCAGAATTTGATTGAAGTTCCTTCCACTTGGCATAAGGAGACACCATCAGCCTGATTGAGAGGGTCATGGTGAGATGGAGCCCGCCAAGGTGGCAGCGCTGAGCTGACCACGCCACGGACCATAGAGTGGGAGCCTTTCCTGCCCCCTTAACCGCAGCTAACATCAAAAGCACTGGTATGGGCTGTCTAACTGGAACCTGAGTTGGTCTTTGTAATTACGATTCTTTTGGGTTTATTTTGCCAGTTCATTATCCACCCCCCTGAAATCAGGCCTCCCAAATTTAGCAGGTGCTGGGGAGGACCCCAGGGAGTGGCTTGTGGGGGCTAGCTGGTGAAACTACCCTTTCCTTTCTGTTCTATGAGTGTGATGGTGTTTGAGAAATATGGGGCTATGGTTCAGGCGCACTTCACATGTGCAAAGATGGAGAAAGCACTCACCTGCACATTTAGGCTCAGAATATTGATTGAAACATTTTGAAATATCAAAAATAAAATGTTATTTTTAAAGTTTCTCTGAGATTTCGCTTAAGTTTTGGTAGATATTCTTAAATTTTAGTGACCTCAGTTTGGGAATTCAGTAAGCC**A**AATATTGTATCCTTATTATTAGTTATATAGAACTATGCCTTAGACTTTGTTAGAAACTTCTGCTTCAGCTTGACTGACTCATTTTCCATTTCTGGTTGTACAAAATGAACTGACACTTTAATGCTGTGGCCACCTTTAAATAAAGTACAATGTGACAAAAAAATACAAA | |
| >rs579501 C  TCCTCAAGACTAGAAAGCATGAAGCAGGGGAGGCGTTTTGAAAGCTTAAGGACAAAAGACCATGGGCCTGGATGGCTGATTCCGGGATCGGCATCGTAATTTGTTGAGAAGGAGGCCGTGCTGTGCTGCCAGTTATTAATGGGTTTAATCGGTTGATACACAGCCCTACTGGCCTAACCAGTAGCCCAGGGCCCGAGAGGATTTGCAGGTCGTGTCAGAATTTGATTGAAGTTCCTTCCACTTGGCATAAGGAGACACCATCAGCCTGATTGAGAGGGTCATGGTGAGATGGAGCCCGCCAAGGTGGCAGCGCTGAGCTGACCACGCCACGGACCATAGAGTGGGAGCCTTTCCTGCCCCCTTAACCGCAGCTAACATCAAAAGCACTGGTATGGGCTGTCTAACTGGAACCTGAGTTGGTCTTTGTAATTACGATTCTTTTGGGTTTATTTTGCCAGTTCATTATCCACCCCCCTGAAATCAGGCCTCCCAAATTTAGCAGGTGCTGGGGAGGACCCCAGGGAGTGGCTTGTGGGGGCTAGCTGGTGAAACTACCCTTTCCTTTCTGTTCTATGAGTGTGATGGTGTTTGAGAAATATGGGGCTATGGTTCAGGCGCACTTCACATGTGCAAAGATGGAGAAAGCACTCACCTGCACATTTAGGCTCAGAATATTGATTGAAACATTTTGAAATATCAAAAATAAAATGTTATTTTTAAAGTTTCTCTGAGATTTCGCTTAAGTTTTGGTAGATATTCTTAAATTTTAGTGACCTCAGTTTGGGAATTCAGTAAGCC**C**AATATTGTATCCTTATTATTAGTTATATAGAACTATGCCTTAGACTTTGTTAGAAACTTCTGCTTCAGCTTGACTGACTCATTTTCCATTTCTGGTTGTACAAAATGAACTGACACTTTAATGCTGTGGCCACCTTTAAATAAAGTACAATGTGACAAAAAAATACAAA | |

Supplement table 2: Predication target binding protein with hsa-miR-27a-5p in miRDB database (Score > 90).

| Target Gene Symbol | Target Rank | Target Score | miRNA Name | Gene Description |
| --- | --- | --- | --- | --- |
| RFK | 1 | 97 | hsa-miR-27a-5p | riboflavin kinase |
| LTBP1 | 2 | 97 | hsa-miR-27a-5p | latent transforming growth factor beta binding protein 1 |
| INO80D | 3 | 95 | hsa-miR-27a-5p | INO80 complex subunit D |
| BTF3 | 4 | 95 | hsa-miR-27a-5p | basic transcription factor 3 |
| IFI30 | 5 | 94 | hsa-miR-27a-5p | IFI30, lysosomal thiol reductase |
| HECW2 | 6 | 94 | hsa-miR-27a-5p | HECT, C2 and WW domain containing E3 ubiquitin protein ligase 2 |
| IL2 | 7 | 94 | hsa-miR-27a-5p | interleukin 2 |
| ADCY1 | 8 | 92 | hsa-miR-27a-5p | adenylate cyclase 1 |
| EIF5 | 9 | 91 | hsa-miR-27a-5p | eukaryotic translation initiation factor 5 |
| NPM1 | 10 | 91 | hsa-miR-27a-5p | nucleophosmin 1 |
| ANTXR1 | 11 | 91 | hsa-miR-27a-5p | ANTXR cell adhesion molecule 1 |
| SLC23A2 | 12 | 91 | hsa-miR-27a-5p | solute carrier family 23-member 2 |
| **GSDMA** | 13 | 90 | hsa-miR-27a-5p | gasdermin A |
